# Perinatal Health Disparities Between Roma and Non-Roma Populations: A Systematic Review

**DOI:** 10.3390/epidemiologia6040082

**Published:** 2025-11-30

**Authors:** Afroditi Dimogerontaki, Nikoletta Iacovidou, Styliani Paliatsiou, Paraskevi Volaki, Theodoros Xanthos, Ioannis Panagiotopoulos, Zoi Iliodromiti, Theodora Boutsikou, Rozeta Sokou

**Affiliations:** 1 Pediatrics Department, Corinth General Hospital, 201 00 Corinth, Greece; a.dimogerontaki@gmail.com; 2 Neonatal Department, Aretaieio Hospital, National and Kapodistrian University of Athens, 115 28 Athens, Greece; niakobid@med.uoa.gr (N.I.); stpaliatsiou@yahoo.gr (S.P.); v.volaki@hotmail.com (P.V.); ziliodromiti@med.uoa.gr (Z.I.); theobtsk@med.uoa.gr (T.B.); 3 School of Health Science, University of West Attica, 122 43 Athens, Greece; theodorosxanthos@yahoo.com; 4 Cardiology Department, Onassis Hospital, 176 74 Athens, Greece; jpanagiotopoulos@outlook.com

**Keywords:** Roma, ethnic minorities, health disparities, perinatal outcomes, maternal health, neonatal health, preterm birth, socioeconomic factors, prenatal care

## Abstract

Background: Women from Roma communities face considerable health inequalities, primarily due to limited access to healthcare systems, alongside broader social and structural disadvantages. Among Roma women these disparities are reflected in poorer perinatal outcomes when compared to non-Roma populations. This systematic review aims at: (a) exploring disparities in neonatal health outcomes between Roma and non-Roma populations in relation to maternal factors such as health status, lifestyle, and education; (b) summarizing key perinatal characteristics in these groups; (c) assessing the influence of prenatal care on neonatal outcomes. Comprehending these disparities is crucial for guiding effective interventions and promoting health equity. Methods: A systematic literature review was conducted in major databases, such as PubMed and Scopus, to identify studies published up to 2025. The eligible studies focused on observational research that compared perinatal outcomes, including preterm birth, low birth weight (LBW), stillbirth, and neonatal mortality, between Roma and non-Roma populations. The potential discrepancies between these populations are thoroughly discussed in the review. Results: A comprehensive search yielded a total of 157 studies. After meticulous screening, 48 relevant studies were identified, reporting substantial health disparities between Roma and non-Roma mothers and their newborns. Roma populations exhibited significantly increased rates of preterm birth, LBW, and neonatal mortality vs. non-Roma populations. Socioeconomic status, access to prenatal care, maternal education, and systemic discrimination were identified as the primary contributing factors to these disparities. Conclusions: The findings highlight the significant and enduring disparities in perinatal health between Roma and non-Roma populations. In order to effectively address these disparities, it is necessary to have a comprehensive and multi-level strategy that prioritizes the social determinants of health, ensures equitable access to high-quality maternal care, and mitigates actively systemic discrimination. Future research should prioritize the development and rigorous evaluation of targeted interventions to reduce these inequities and improve perinatal outcomes among Roma populations.

## 1. Introduction

Perinatal health is a crucial indicator of the overall well-being of populations, yet substantial disparities persist across diverse ethnic and socioeconomic groups. Among the most marginalized communities globally, the Roma population consistently experiences poorer health outcomes compared to their non-Roma counterparts [1]. These disparities in perinatal health, encompassing higher rates of adverse neonatal effects, underscore the need for targeted research and intervention. Structural inequalities, such as limited access to healthcare services, socioeconomic disadvantage, discrimination, and cultural barriers, contribute to these adverse outcomes [2,3]. Despite growing attention to minority health concerns, the specific perinatal health challenges faced by Roma populations remain under-researched and inadequately addressed in public health initiatives [4].

The Roma population faces social exclusion, poverty, and segregation, leading to limited access to essential services and contributing to adverse maternal and perinatal health outcomes. These challenges were exacerbated during the COVID-19 pandemic due to mobility restrictions and reduced healthcare access [5].

This systematic review with qualitative synthesis aims to present the existing evidence on perinatal health disparities between Roma and non-Roma populations, providing a descriptive analysis of the factors contributing to these disparities and their implications. By consolidating current findings, this study further aims at directing future research into the issue, that would design healthcare policies and practice to promote health equity for Roma mothers and their newborns.

We conducted a systematic review of the published literature to achieve the following objectives: (a) gain a deeper understanding of neonatal health disparities between Roma and non-Roma newborns in relation to maternal health, lifestyle, and education; (b) provide comprehensive data on perinatal characteristics within these subgroups; (c) examine the impact of prenatal care on neonatal outcomes. The PECO framework (Population, Exposure, Comparison, Outcomes) was used to structure the research question and guide evidence selection and synthesis.

## 2. Materials and Methods

### 2.1. Search Strategy–Data Source

This systematic review was conducted from May 2025 to September 2025 and is registered with PROSPERO (ID CRD420251042394). The PubMed and Scopus databases were systematically searched for studies reporting disparities between Roma and non-Roma newborns in terms of birth outcomes related to socioeconomic factors, maternal health status, and prenatal care. The search was limited to studies published in English.

The combination of keywords used in PubMed included: (“Roma” OR “Gypsy” OR “Traveller” OR “Traveler” OR “Romani”) AND (“Infant, Newborn”[MeSH] OR “neonate” OR “newborn”) AND (“Prenatal Care”[MeSH] OR “Maternal Health”[MeSH] OR “Socioeconomic Factors”[MeSH] OR “Perinatal Care”[MeSH] OR “low birth weight” OR “preterm birth”). In Scopus, the search terms were: (“Roma” OR “Gypsy” OR “Traveller” OR “Traveler” OR “Romani”) AND (“infant” OR “newborn” OR “neonate”) AND (“prenatal care” OR “maternal health” OR “socioeconomic factors” OR “perinatal care” OR “low birth weight” OR “preterm birth”).

In order not to omit studies in which the Roma population is referenced with another name, the terms “Traveller”, “Gypsy” and “Romani” were also screened. These terms are frequently found in worldwide bibliography when the Roma population is studied.

To ensure comprehensive coverage of the literature and reduce the risk of missing relevant studies, references of included studies and previous systematic reviews on the topic were manually screened. The methodological quality of all included observational studies was assessed independently by two reviewers using the Newcastle–Ottawa Scale (NOS), with the main quality considerations integrated into the qualitative synthesis.

The search protocol was developed according to PRISMA 2020 recommendations. The complete search strategy—including full database search strings, filters applied, and the exact date of the final search—is available upon request. No additional sources (grey literature, organizational websites, thesis repositories, or conference proceedings) were searched. Duplicate records were removed using an automated deduplication tool, followed by manual verification. No backward or forward citation tracking was performed. A complete PRISMA 2020 flowchart is included to document all steps of identification, screening, eligibility assessment, and study inclusion.

To increase methodological clarity, the research question was structured using the PECO framework: Population (Roma pregnant women and their newborns), Exposure (maternal socioeconomic status, lifestyle behaviors, and access to prenatal care), Comparison (non-Roma populations within the same geographical settings), and Outcomes (birth weight, gestational age, neonatal morbidity and mortality). This framework guided the selection, extraction, and synthesis of evidence.

### 2.2. Eligibility Criteria

Studies included were observational in design (cohort, cross-sectional, or case–control studies) and reported on Roma, Gypsy, Traveller, or Romani populations during the prenatal and perinatal period.

To ensure methodological consistency, the review applied clearly defined inclusion and exclusion criteria. Inclusion criteria required that studies: involved Roma populations explicitly identified through self-identification, census data, or ethnicity registries; included a non-Roma comparison group; reported quantitative perinatal or neonatal outcomes such as preterm birth, low birth weight, stillbirth, neonatal mortality, congenital anomalies, or morbidity indicators; employed observational designs (cohort, case–control, cross-sectional); and were peer-reviewed, full-text articles published in English.

Exclusion criteria encompassed: reviews, editorials, commentaries, conference abstracts, or qualitative studies; studies lacking comparative data between Roma and non-Roma populations; papers that did not provide clearly defined perinatal or neonatal outcomes; studies where Roma ethnicity was not explicitly stated or could not be reliably inferred; and publications not available in English or without accessible full text.

These criteria ensured the selection of studies capable of reliably contributing to the synthesis of perinatal disparities between Roma and non-Roma populations.

### 2.3. Data Extraction and Conflict Resolution

Data extraction and quality assessment were conducted independently by two researchers. Conflicts were resolved through discussion and consensus, or, if necessary, with the involvement of a third researcher.

### 2.4. Data Synthesis and Presentation

Data were recorded in a structured table, including study design, year of publication, sample size, participant characteristics, birth outcomes, maternal health status, prenatal care, socioeconomic factors, and other relevant criteria for the classification of study populations. Potential gaps in the literature were identified, and suggestions for future research were proposed.

## 3. Results

**Figure 1:**
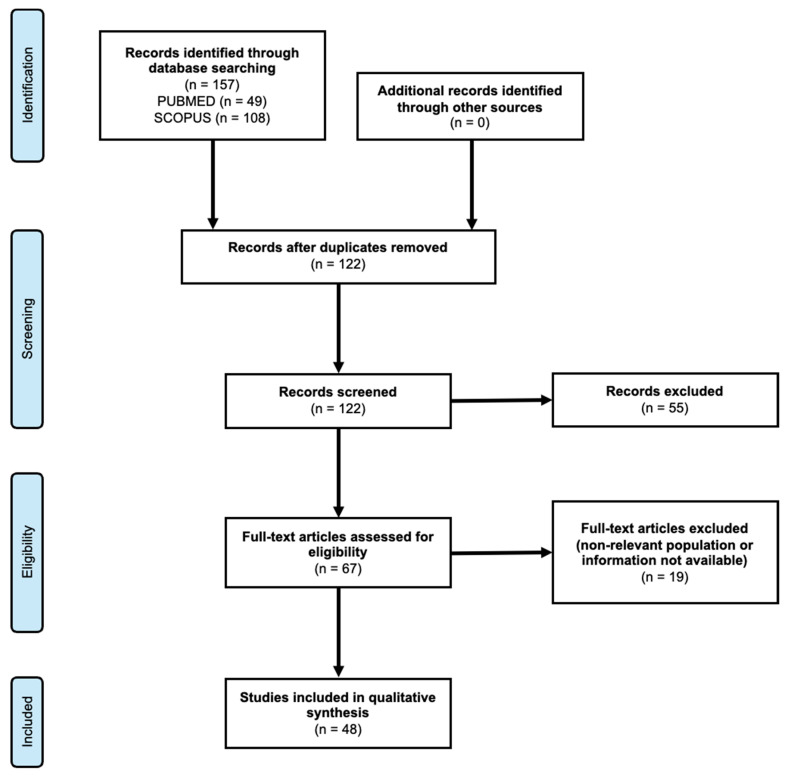
PRISMA flow-chart.

**Figure 2:**
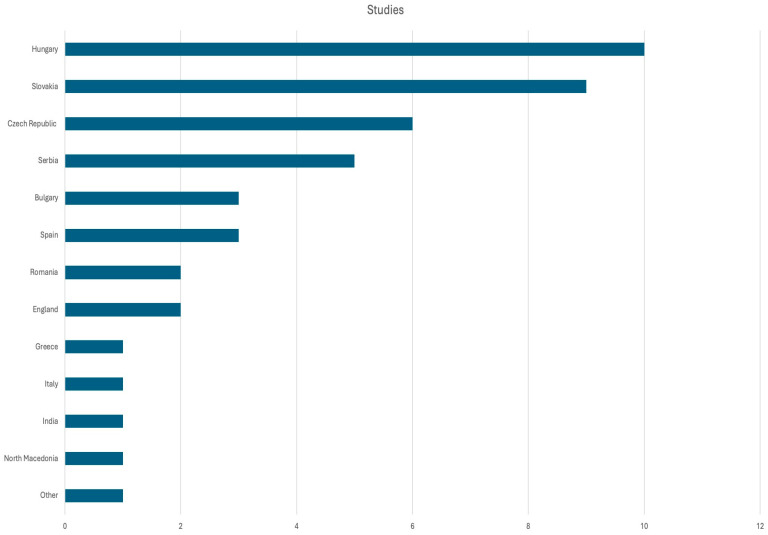
Number of publications identified by country.

From the initial search, 157 relevant studies were identified. A total of 48 eligible studies met the criteria for relevance and were included in the systematic review. The PRISMA flow-chart for the study is presented in Figure 1. It is noteworthy that nearly half of the publications focus on only four countries: Slovakia, Hungary, the Czech Republic, and Serbia. These countries (Figure 2), as reported by the European Parliament, have the largest Roma populations [6].

### 3.1. Birth Weight and Gestational Age

Several studies demonstrated substantial disparities in birth weight (BW). Table 1 provides an overview of the studies examining birth weight and gestational age disparities between Roma and non-Roma populations. The table summarizes the study design, population characteristics, key risk factors, and main findings regarding low birth weight, preterm birth, and fetal growth restriction. A survey conducted by Bobak et al. reported that Roma infants exhibited significantly lower BW, gestational age, and a markedly higher incidence of intrauterine growth restriction (IUGR) [7]. Similar findings were reported by Diabelková J et al. and Balázs et al. [8,9,10], who identified risk factors associated with preterm birth and LBW. The results indicated that Roma mothers, particularly those who were single, uneducated, smoked during pregnancy, or consumed alcohol, were at a higher risk of these conditions [8,9,10]. However, ethnicity was not a significant factor in these outcomes. Factors associated with LBW and preterm birth included being underweight and smoke, usually observed in Roma mothers [11]. Mothers with a low body mass index, limited education, who smoked during pregnancy, of age at birth less than 18 years, who had inadequate housing, and were not consuming sufficient amount of fruits or dairy products, influenced the neonate’s BW in a statistically significant manner [12]. According to the Cohort ’18–Growing Up in Hungary study, 5.9% of women who gave birth to a single child between April 2018, and April 2019 had a LBW infant. While bivariate analysis showed that lower income, Roma ethnicity, smoking during pregnancy, and living in a less developed region increased the likelihood of LBW, in multivariate analysis only maternal smoking and low maternal education remained significant risk factors [13]. Evidence from Roma settlements suggests that children born with LBW face significant nutritional deficits [14,15]. Grbic d. et al. reported that women with LBW newborns were significantly more likely to reside in settlements predominantly inhabited by Roma [16]. LBW Roma newborns were also confirmed by other studies, which investigated the health of Roma children [17,18,19]. Dejmek et al. reported that the prevalence of LBW neonates was 23.6% among Roma births [20]. Infants born to Roma mothers exhibited LBW and shorter length, and pregnancy duration was approximately one week shorter [21]. Both lower BW and smaller head circumference were independently associated with Roma ethnicity and smoking, as validated by Walfisch et al. [22].

### 3.2. Infant Mortality and Morbidity

Rosicova et al. [23] investigated the influence of educational level, unemployment status, and income on perinatal and infant mortality. Their analysis identified that the only factor with a statistically significant effect was the proportion of the Roma population [23]. Bosakova et al. linked socioeconomic disadvantage and ethnicity to increased infant mortality, and concluded that the Roma population had a higher mortality rate, especially during the COVID-19 pandemic. Factors such as limited elementary education, long-term unemployment, and Roma ethnicity contributed to this fact [2]. On the contrary, David E. Odd et al. in a study conducted in England between 2019 and 2022, found that all infants had an equal risk of mortality, regardless of racial or ethnic background [24].

In terms of morbidity, several syndromes and conditions were reported with increased frequency among Roma infants. Table 2 summarizes studies reporting neonatal morbidity patterns in Roma populations, including congenital anomalies, genetic syndromes, infectious disease outcomes, and other clinical conditions. For example, isolated anorectal malformations were more prevalent among younger mothers with lower socioeconomic status—factors commonly observed within Roma communities [25]. Mazurova et al. [26] reported fatal neonatal nephrocutaneous syndrome in 18 newborns with epidermal growth factor receptor (EGFR) deficiency—a multisystemic disorder caused by a homozygous mutation and associated with poor prognosis. All affected infants in the study were of Roma origin, with most families originating from Eastern Slovakia, strongly suggesting a ‘founder effect’ [26]. This finding indicates that the mutation may represent an underrecognized cause of congenital illness within the Roma population [27]. Another study, on isolated primary glaucoma, revealed a higher incidence in the Hungarian Roma population, which is associated with their inbreeding and the potential ‘founder effect’ of a gene mutation [28]. Neonatal abstinence syndrome has been observed to increase among children of young Roma mothers, as reported by Pèrez Bescos L. et al. [29] Roma communities often face socioeconomic disadvantage, limited access to healthcare services, and social marginalization, which may contribute to higher rates of substance use and reduced opportunities for early prenatal care. Young mothers in particular may have lower health literacy, limited support systems, and higher exposure to risk behaviors, including drug use. Moreover, barriers such as discrimination, poverty, and mistrust of health institutions can delay or prevent these mothers from receiving adequate treatment for substance dependence during pregnancy. All these factors increase the likelihood of intrauterine drug exposure, and therefore the incidence of NAS among their newborns [29]. The Roma population experienced a higher prevalence of isolated anopthalmia and microphtalmia at birth due to the interaction of the inbreeding effect and low socioeconomic status. Roma mothers are often younger, more frequently unmarried, and come from lower socioeconomic backgrounds. Many were employed primarily in agriculture, while unemployment was notably more common compared to the general population. These circumstances likely accounted for the reduced use of pregnancy supplements such as folic acid and iron. In addition, an unusually high proportion of mothers did not respond to the surveys. Therefore, it can be suggested that Hungarian Roma infants face an increased risk of IAM [30]. A correlation between the somatic developmental status of newborns and sociodemographic data indicated that the physical development of Roma infants is significantly inferior to that of the national average [31].

During the measles outbreak in Bulgaria, infants from Roma communities were disproportionately affected, demonstrating inadequate vaccination and maternal education of this population [32]. Hepatitis B infection is more prevalent among immigrant and Roma women compared to native women, affecting newborns as well [33]. Finally, despite that the risk of pregnancy with minimal or no prenatal care was higher among non-native and Roma populations, the differences in hospital admissions are not attributed to imported or genetic diseases but are likely due to variations in the social and cultural environment during pregnancy in these populations [34].

### 3.3. Vaccination

Vaccination is another crucial aspect of Roma health. Moukagni M. et al. [18] confirmed that the vaccination status of Roma newborns and children was inadequate, leaving them vulnerable to vaccine-preventable diseases such as measles and pertussis. This gap is linked to socioeconomic barriers, limited access to healthcare, and mistrust toward health services. The authors highlighted the need for targeted interventions, including mobile vaccination units and culturally adapted health education. These disparities do not appear to be fully attributable to their lower socioeconomic status, as the probability of vaccination increases with access to healthcare services, particularly when Roma individuals have a doctor to consult when necessary [18,35]. In contrast, a study examining the acceptance of a postpartum influenza vaccination in Greece found that Roma origin was a statistically significant factor associated with increased vaccination rates. Roma mothers frequently face difficulties to access health-care services because of economic, cultural or language issues, thus the on-site and free-of-charge delivery of influenza vaccination may eliminate the barrier of cost [36].

### 3.4. Breastfeeding

As for breastfeeding, the evidence is more encouraging. The prevalence of exclusive breastfeeding was nearly identical in non-Roma and Roma mothers. However, it is noteworthy that in the Roma population, there was no single woman who attended childbirth preparation classes [37]. Research shows that Gypsy, Traveller, and Roma communities often rely on traditional practices and intra-community support for infant feeding, with Roma mothers in particular maintaining breastfeeding and timely weaning. However, when professional support was sought, healthcare provision was frequently perceived as inadequate, underscoring the need for culturally sensitive health promotion strategies [38]. Data indicates that breastfeeding rates among Roma women are very low, with formula feeding being the predominant practice. However, their generally neutral attitudes toward infant feeding suggest that targeted interventions and culturally adapted health promotion could positively influence breastfeeding initiation in this community [39].

### 3.5. Lifestyle, Socioeconomic Factors and Maternal Education

One of the most significant factors associated with the health and outcomes of newborns is maternal lifestyle. Maternal education is proportional to LBW and it is the single most important factor explaining the poor birth outcomes, and this is confirmed in multiple studies [7,13,40,41]. Gender, living district, maternal age, and number of pregnancies contributed only marginally to LBW. Balázs et al. aimed at explaining LBW, and revealed that lifestyle habits, including primary maternal education, marital status (single), number of prenatal core visits, and preterm birth, are to be attributed [11]. Joubert K. et al. confirmed that Roma newborns consistently tend to be smaller, and this difference is attributed to the circumstances under which Roma live, which often involve poverty, and have poorer maternal education [41]. Another study revealed that the characteristics of the Roma population, including their lifestyle and irregular medical follow-up, result in maternal and neonatal morbidity [42,43]. Poor living conditions, low educational levels, low employment rates, and barriers to access healthcare services are also risk factors for the health status of Roma mothers and children [44].

An interesting fact is that 40% of Roma women admitted smoking during pregnancy. Roman ethnicity and maternal smoking were significantly associated with a history of abortions, IUGR and preterm births [20,21,22,45]. Significantly decreased Vitamin C levels were observed in Roma mothers and their babies, which may be attributed to unfavorable diets and smoking habits. Pavuk et al. [46] confirmed that the percentage of no smoking women was significantly lower among Roma women. These women were more likely to complete only primary education or attend trade schools [46].

Other hazardous environmental factors are also more likely to be described among Roma population, and their infants are often exposed to dangerous elements. For example, in eastern Slovakia, exposure to polychlorinated biphenyls (PCB) result in LBW in Roma boys [47]. Not only that, but even if living conditions are acceptable, many Roma children may be at risk of undernutrition or antenatal care [14]. Stoica et al., reported that all Roma mothers and almost all their newborns were deficient in Vitamin D or had insufficiency [48]. Adding to these drawbacks, high maternal stress, as defined by high hair cortisol concentrations, further deteriorate mother and child mental and physical health [49].

Regarding maternal age at childbirth, there was no association with either nutritional or growth outcomes of Roma children. Instead, child characteristics (male sex, LBW, unwanted, younger age) and maternal characteristics (short birth spacing, higher parity, low socioeconomic status) were associated with children’s malnutrition [50]. Similar results were reported in another study, confirming that teenage pregnancies in Roma women may not always be associated with poorer health status [51]. Roma women gave birth at a younger age (before 20 years old), had a statistically significant higher occurrence of preterm births, and delivered more newborns with LBW, possibly attributed to unknown cofounders. However, it should be noted that numerous studies evaluated obstetric and neonatal outcomes in relation to maternal age, placing particular emphasis on pregnancies in women under 17 years and over 35 years [46]. Occasionally, studies report higher rate of premature delivery, anemia, preeclampsia-eclampsia, and overweight in younger Roma women [52].

A prenatal and birth care study of Roma women showed that prenatal care is lacking in Roma women, but this did not imply worse neonatal health outcomes. These findings suggest that, in Spain, Roma women experience higher levels of social inclusion and a comparatively better socioeconomic position than in other countries, where Roma communities often reside in peripheral settlements marked by poor infrastructure, inadequate hygiene and housing conditions, and deeper social exclusion [53]. This fact seems to be paradoxical, as Szabó et al. showed that discrimination exists in Roma women during childbirth and Roma mothers have lower rate of cesarean sections due to fewer planed interventions, are less likely to have a birth attended by a private obstetrician or have a family member present at birth [54]. These significant disadvantages in maternity care are also underlined by other studies and overall seem to have a negative impact on maternal and neonatal health. They are attributed to the Roma ethnicity, socio-economic and regional factors. Mitrut et al. proved that when a public health program in Romania was conducted that targeted Roma people, it led to a large increase in prenatal care rate. However, no improvements were observed in children’s health at birth [55]. For this reason, Janevic et al. imply that improving Roma socioeconomic status and supporting their rights as human beings may lead to a decrease in neonatal adverse effects, such as LBW [3].

## 4. Discussion

This systematic review consolidates findings from 48 studies on perinatal and neonatal health disparities affecting Roma populations, revealing that Roma newborns consistently face worse outcomes compared to their non-Roma counterparts. Increased incidence of LBW, preterm birth, and neonatal morbidity among Roma infants are well documented. However, these disparities are not only attributed to ethnicity but are closely linked to a range of socioeconomic and structural determinants.

Maternal education emerged as one of the most significant predictors of neonatal health outcomes. Studies showed that Roma women with lower educational attainment are more likely to have LBW or preterm infants. This finding emphasizes the importance of improving educational opportunities for Roma women as a strategy to enhance maternal and child health.

Risk behaviors such as smoking during pregnancy, poor diet, and alcohol consumption were frequently observed among Roma women, compounding the risk of adverse outcomes. For example, studies reported significantly lower smoking abstinence among Roma women, along with reduced Vitamin C levels, which are linked to poor neonatal nutrition and immunity.

Several congenital and genetic conditions, including primary glaucoma and neonatal nephrocutaneous syndrome, were more prevalent in Roma populations, possibly due to higher consanguinity rates and limited access to prenatal screening. These findings underline the need for improved access to early diagnostic care and preventive genetic counseling in marginalized communities.

Vaccination disparities were another critical issue. Roma children and newborns often had inadequate immunization coverage, which does not appear to be fully explained by socioeconomic factors on itself. Instead, access to healthcare and trust in medical providers played an influential role, suggesting the need for inclusive, trust-building interventions.

Some results offered a more refined perspective on the issue. For instance, one study found that exclusive breastfeeding rates were nearly equal between Roma and non-Roma women. This may reflect cultural practices or economic necessity, as formula milk is less accessible to low-income families. However, the same study reported that none of the Roma women had attended childbirth education classes, indicating systemic exclusion from preparatory health services.

Few studies reported no significant difference in neonatal mortality when controlling for socioeconomic variables, reinforcing the idea that ethnicity itself is not the primary determinant of poor outcomes, but rather, ethnicity acts as a proxy for deeper structural inequities.

Discrimination in healthcare access and delivery was widely documented. Roma women were less likely to deliver by cesarean sections, be attended by private obstetricians, or have a support person present during birth. These findings were echoed across several studies and point to institutional barriers within healthcare systems.

While this review synthesizes a wide range of observational studies, it is important to acknowledge potential limitations. These include publication bias, as studies showing significant disparities may be more likely to be published, and regional concentration bias, with a substantial proportion of the literature focused on Central and Eastern Europe. Additionally, methodological heterogeneity among studies, such as variations in how ethnicity is classified, the timing and type of prenatal care assessed, and differing healthcare systems, may influence the comparability and generalizability of results. Lastly, only a very small number of potentially relevant studies were published in languages other than English; therefore, although language bias cannot be entirely excluded, its impact on the present review is likely minimal.

## 5. Conclusions

Τhis review confirms that Roma populations face disproportionate risks in perinatal and neonatal health, largely driven by social determinants such as low maternal education, poverty, limited prenatal care, and systemic discrimination. Risk behaviors such as smoking and poor nutrition further contribute to adverse outcomes like LBW and preterm birth. While some congenital conditions may have genetic underpinnings, the lack of adequate prenatal screening and healthcare access plays a major role in their prevalence and severity.

This review has several limitations. Most included studies originate from Central and Eastern Europe, introducing regional bias and limiting generalizability to other Roma communities. Restricting the search to English-language publications may have led to language bias. Additionally, considerable heterogeneity across studies—particularly in design, outcome definitions, and methods of identifying Roma ethnicity—reduced comparability and prevented meta-analysis. These factors should be considered when interpreting the findings.

Improving perinatal outcomes in Roma communities requires a multifaceted, equity-focused approach that extends beyond healthcare. Policies should prioritize maternal education, housing, and anti-discrimination practices, while ensuring culturally appropriate and accessible prenatal care. Interventions must be designed in collaboration with Roma communities to foster trust and effectiveness. Longitudinal studies are also needed to assess the lasting impact of early-life disparities and to guide targeted public health efforts.

## Figures and Tables

**Table 1:** Overview of Studies on BW and Gestational Age in Roma Communities.

Author(s)	Country	Study Design	Aim	Population	Main Finding
Bobak M, Dejmek J, Solansky I, Sram RJ (2005) [7]	Czech Republic	Population-based study	To explore adverse birth outcomes among Roma women and potential explanations	Roma and non-Roma mothers	Roma women had higher risk of LBW and preterm births; partly explained by socioeconomic and lifestyle factors
Diabelková J et al. (2018) [8]	Slovakia	Comparative study (Roma vs. non-Roma)	To identify risk factors for preterm birth and LBW	Roma and non-Roma mothers	Roma mothers had significantly higher rates of LBW and preterm births; maternal education and socioeconomic status were strong predictors
Diabelková J et al. (2022) [9]	Slovakia	Cross-sectional (risk factor analysis)	To evaluate risk factors associated with LBW	Mothers delivering in Slovakia	Low education, Roma ethnicity, smoking and poor socioeconomic conditions increased LBW risk
Balázs P et al. (2014) [10]	Hungary	Cross-sectional	To analyze biomedical and socioeconomic determinants of BW in Roma neonates	Roma newborns in Hungary	Socioeconomic disadvantages strongly affected Roma BW outcomes
Balázs P et al. (2013, Eur J Public Health) [11]	Hungary	Population-based study	To assess risk factors of preterm and LBW births	Roma and non-Roma mothers	Roma mothers had higher incidence of LBW and preterm; low maternal education and poor living conditions were key factors
Balázs P et al. (2014, Cent Eur J Public Health) [12]	Hungary	Population-based study	To compare BW differences between Roma and non-Roma neonates	Roma vs. non-Roma neonates	Roma newborns had significantly lower mean BW; implications for health inequalities
Szabó L, Boros J (2023) [13]	Hungary	Birth cohort (Cohort ’18)	To analyze socioeconomic differences in LBW	Hungarian birth cohort (2018–2019)	LBW prevalence 5.9% overall; low education and maternal smoking main predictors; Roma ethnicity significant only in bivariate analysis
Čvorović J (2023) [14]	Serbia	Field study in Roma settlements	To examine growth penalties of unwanted children	Roma children in poor settlements	Unwanted Roma children had higher risk of growth restrictions and LBW
Majdan M et al. (2018) [15]	Slovakia	Cross-sectional (2009–2013)	To compare birthweight patterns in rural areas with/without Roma communities	Birth records from rural municipalities	Municipalities with Roma communities showed higher LBW prevalence
Grbic D et al. (2024) [16]	Western Balkans	Survey analysis (MICS data)	To investigate LBW risk factors in low-income groups	Mothers/infants in low-income households	Low maternal education, poverty, and Roma background linked with higher LBW
Bereczkei T et al. (2000) [17]	Hungary	Evolutionary/anthropological study	To analyze LBW, maternal spacing and reproductive decisions	Hungarian mothers (including Roma)	LBW associated with maternal reproductive strategies and future fertility decisions
Pelzer Moukagni M et al. (2011) [18]	France (Lille)	Retrospective perinatal study	To evaluate perinatal care and child health in Roma children up to age 6	Roma children in Lille	Roma infants had poorer perinatal outcomes, including higher LBW
Papp C et al. (1991) [19]	Hungary	Retrospective (fetal growth study)	To analyze fetal growth variations in 1988–89	Hungarian births	Reported variations in growth and LBW; socioeconomic factors relevant
Dejmek J et al. (1996) [20]	Czech Republic	Observational study	To study environment, lifestyle, and pregnancy outcome	Czech mothers (including Roma)	Smoking, low SES, and Roma ethnicity linked to LBW
Rambousková J et al. (2009) [21]	Czech Republic	Cross-sectional study	To assess maternal/infant health behaviors, nutrition, and anthropometry	Roma and non-Roma mothers & infants	Roma infants had poorer nutrition and higher LBW prevalence
Walfisch A et al. (2013) [22]	North Macedonia	Observational	To examine link between smoking and fetal growth restriction in Roma	Roma pregnant women	Maternal smoking strongly associated with fetal growth restriction and LBW

**Table 2:** Studies on Neonatal Morbidity.

Author(s)	Country	Study Design	Aim	Population	Main Finding
Vermes G, László D, Czeizel AE, Ács N (2016) [25]	Hungary	Population-based case–control study	To evaluate birth outcomes in patients with isolated anorectal malformations	Newborns with isolated anorectal malformations and controls	Increased risk of adverse birth outcomes among affected infants compared to controls
Mazurova S et al. (2020) [26]	Czech Republic	Case series	To describe fatal neonatal nephrocutaneous syndrome with EGFR deficiency	18 Roma children with EGFR deficiency	EGFR deficiency caused fatal neonatal syndrome in all cases; highlighted genetic vulnerability in Roma population
Ganetzky R et al. (2015) [27]	USA	Genetic/molecular study	To characterize lethal epithelial dysfunction syndrome caused by EGFR mutations	Patients with EGFR mutations	EGFR mutations lead to progeroid features and lethal epithelial dysfunction; molecular basis identified
Vogt G, Horváth-Puhó E, Czeizel AE (2006) [28]	Hungary	Population-based case–control study	To study isolated primary congenital glaucoma	Newborns with congenital glaucoma and matched controls	Identified potential risk factors and higher prevalence among affected infants
Pérez-Bescos L et al. (1993) [29]	Mexico	Clinical/epidemiological study	To examine neonatal abstinence syndrome	Newborns affected by neonatal abstinence syndrome	Characterized clinical features and epidemiology; highlighted importance of maternal drug exposure
Vogt G, Puhó E, Czeizel AE (2005) [30]	Hungary	Population-based case–control study	To investigate isolated anophthalmia and microphthalmia	Newborns with anophthalmia/microphthalmia and controls	Risk factors identified; higher prevalence in affected infants, useful for epidemiological insights
Joubert K (1990) [31]	Belgium	Observational study	To correlate newborn somatic developmental status with sociodemographic data	Newborn infants	Showed associations between birth anthropometrics and sociodemographic variables
Lim TA et al. (2013) [32]	Bulgaria	Outbreak investigation	To study risk factors for medical complications during measles outbreak	Infants born to mothers with varying educational levels	Poor maternal education associated with higher risk of medical complications in infants
Mur Sierra A et al. (2010) [33]	Spain	Retrospective study	To assess neonatal repercussions of immigration	Newborns of immigrant vs. native mothers in Spain	Immigrant neonates had higher rates of hospital admission and adverse outcomes in both periods studied
Puig Sola C et al. (2008) [34]	Spain	Cross-sectional hospital-based study	To evaluate neonatal hospital admissions by ethnicity and parental origin	Newborns in urban Barcelona	Ethnic minority and immigrant newborns had higher hospital admission rates compared to local-born infants

## Data Availability

The original data presented in the study are openly available in PubMed and Scopus databases.
